# Loss of compensatory pro-survival and anti-apoptotic modulator, IKKε, sensitizes ovarian cancer cells to CHEK1 loss through an increased level of p21

**DOI:** 10.18632/oncotarget.2665

**Published:** 2014-11-15

**Authors:** Marianne K. Kim, Dong J. Min, George Wright, Ian Goldlust, Christina M. Annunziata

**Affiliations:** ^1^ Women's Malignancies Branch, Center for Cancer Research, National Cancer Institute, Bethesda, MD 20892; ^2^ Transgenic Oncogenic and Genomics Section, Center for Cancer Research, National Cancer Institute, Bethesda, MD 20892; ^3^ Biometrics Research Branch, Center for Cancer Research, National Cancer Institute, Bethesda, MD 20892; ^4^ NIH Chemical Genomics Center, Division of Preclinical Innovation, National Center for Advancing Translational Sciences, Bethesda, MD 20892

**Keywords:** shRNAs, therapeutic targets, IKKε, CHEK1, p21, ovarian cancer

## Abstract

Ovarian cancer (OC) is extremely heterogeneous, implying that therapeutic strategies should be specifically designed based on molecular characteristics of an individual's tumor. Previously, we showed that IKKε promotes invasion and metastasis in a subset of OCs. Here, we identified CHEK1 as an IKKε-dependent lethal gene from shRNA kinome library screen. In subsequent pharmacological intervention studies, the co-inhibition of IKKε and CHEK1 was more effective in killing OC cells than single treatment. At the molecular level, co-inhibition dramatically decreased pro-survival proteins, but increased proteins involved in DNA damage and apoptosis. IKKε-knockdown increased p21 levels, while overexpression of wild-type IKKε, but not a kinase dead IKKε mutant decreased p21 levels. We further demonstrated that the depletion of p21 rendered OC cells more resistant to cell death induced by co-inhibition of IKKε and CHEK1. In conclusion, we revealed a novel interplay between IKKε, CHEK1 and p21 signaling in survival of OC. Our study provides a rationale for the clinical development of specific IKKε inhibitor and for usage of IKKε as an exploratory marker for resistance to CHEK1 inhibitors in the clinic. The interplay provides one potential explanation as to why very few clinical responses were achieved in patients treated with single-agent CHEK1 inhibitors.

## INTRODUCTION

Ovarian cancer (OC) is a heterogeneous disease, made up of several histological subtypes with different biology [1]. The high-grade serous histologic subtype has recently been shown to be a disease of DNA repair, with a common feature of genomic disarray [2]. Defective DNA repair processes are characteristic of the hereditary loss-of-function BRCA mutations, but also appear to be an underlying feature of sporadic ovarian cancers. This feature makes most OCs initially responsive to platinum-based chemotherapy. Most cancers, however, eventually relapse and metastasize, becoming resistant and refractory to standard chemotherapy. Therefore, additional insight is needed regarding the molecular pathways driving recurrent and metastatic OC.

IKKε was previously identified as an oncogene in breast cancer [3] and was associated with poor clinical outcome in OC [4]. We previously showed that IKKε expression was significantly higher in metastatic tumors compared to primary tumors, promoted tumor invasion and metastasis, while its loss moderately decreased cellular proliferation [5]. Therefore, we screened shRNA library to identify IKKε-dependent lethal genes to uncover co-dependent modulator(s) cooperating with IKKε in promoting OC survival and progression.

The concept of synthetic lethality was first utilized in yeast, where the mutation of two individual genes did not affect cell growth, but absence of function in both genes was lethal. This approach was recently applied to cancer cells to understand the biology of selected signaling pathways of interest [6]. Classically, such screening is performed in isogenic models of matched cell line pairs, by mutating one gene at a time. Unfortunately, the procedure of establishing and isolating isogenic cell lines can be arduous. And despite this laborious work, target(s) identified in one specific isogenic cell line pair may not be valid in other cell types or under different experimental conditions. Conventional sensitization screens utilize a small molecule inhibitor in combination with a comprehensive shRNA library to identify genes that are lethal in the presence of the inhibitor but not in its absence [7]. However, a highly specific small molecule inhibitor of IKKε is not readily available for use as a tool compound to study and target IKKε in metastatic ovarian tumors with a high level of IKKε. Therefore, we developed and optimized a rapid and robust dual shRNA technique to perform an IKKε-dependent lethality screen.

Here, we show a novel interplay between IKKε, CHEK1 and p21 to propagate OC cells, via a mechanism involving cell cycle regulation and pro-survival signaling, demonstrating that IKKε exerts anti-apoptotic and pro-survival functions via suppression of p21, while CHEK1 repairs intrinsic DNA damage for survival.

## RESULTS

### Kinome shRNA library screen and target identification in IKKε-depleted OC cells

**Figure 1:**
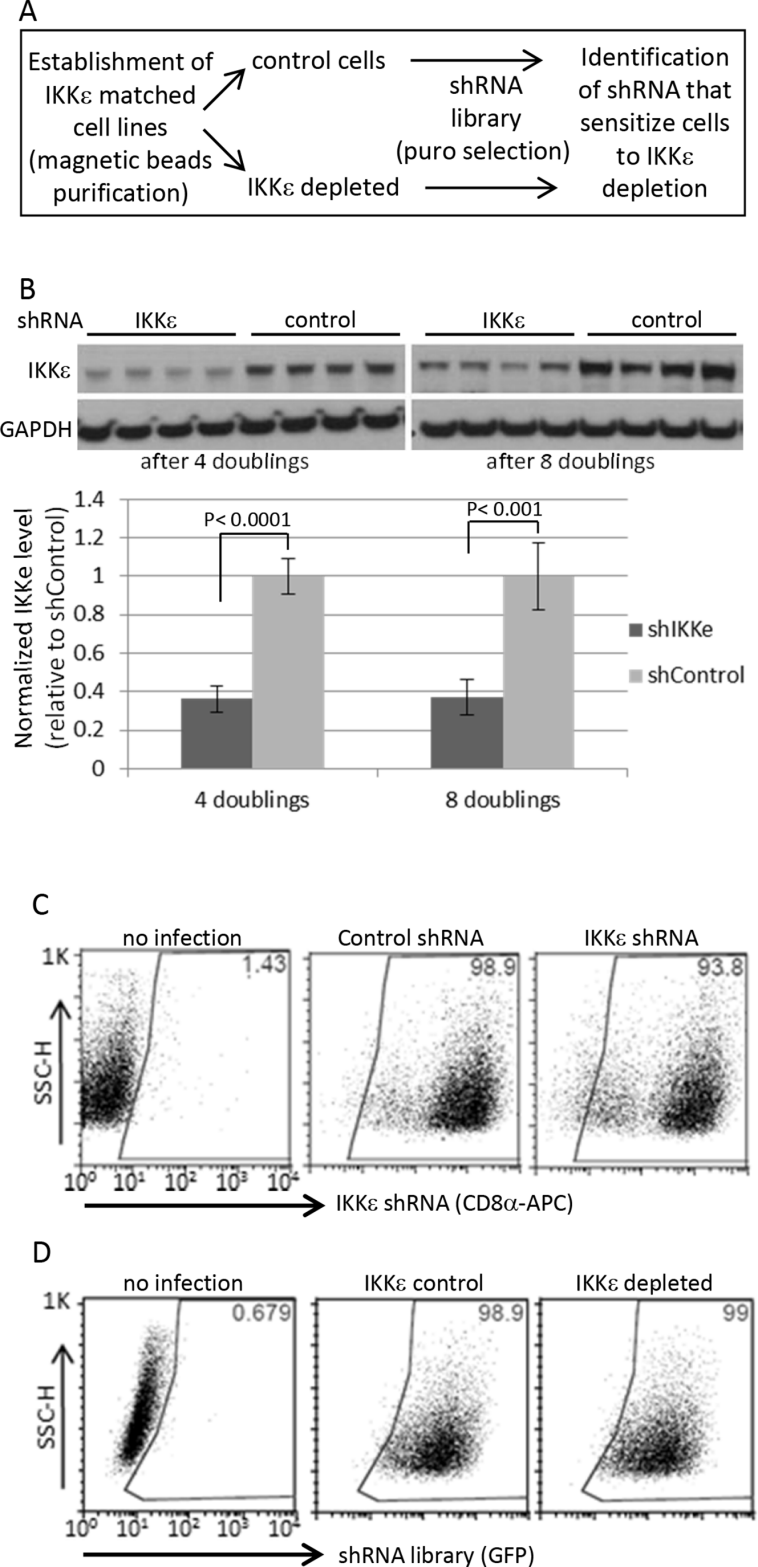
Human kinome shRNA screening **(A)** Dual shRNA screening procedure **(B)** The magnetic beads-based purified cells followed by shRNA library infection were maintained for indicated times upon completion of puromycin selection for 4 days, and then harvested for Western blotting in four biological replicates. For quantification, the signals were quantified by ImageJ software. IKKε expression was normalized by GAPDH level. The statistical significance was determined by 2-sided t-test. **(C)** The purity of Ovcar5 CD8α-positive cells (IKKε-matched cell line pairs) was measured by FACS at 13 days after beads purification. **(D)** The shRNA library vectors co-expressing GFP were introduced into the pseudo-isogenic IKKε-control and -depleted cell line pair. The purity of shRNA library in Ovcar5 cells was measured by GFP expression 2 days after completion of puromycin selection.

**Figure 2:**
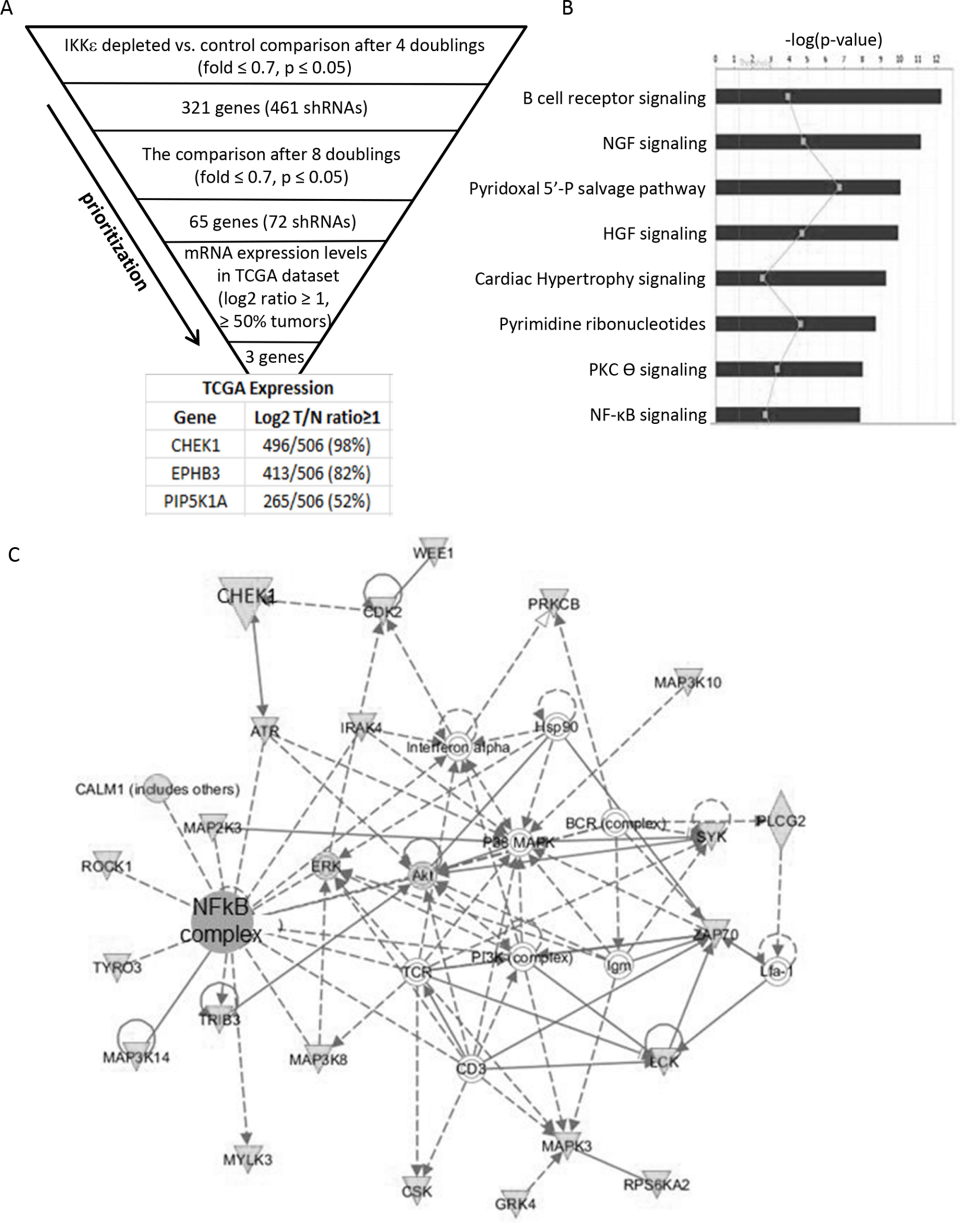
Identification of candidate genes **(A)** The criteria and prioritization steps to identify IKKε-dependent lethal genes are shown. Sixty-five genes identified at two different time points were queried in the TCGA database to examine their expression levels in ovarian cancers, identifying 3 genes with a cut-off of a 2 fold overexpression in 50% of tumors relative to normal controls. **(B)** Sixty five candidate genes were uploaded onto Ingenuity Pathway Analysis and top canonical pathways with significant *p* values were identified and shown in a bar graph. **(C)** The most significant network is shown.

We previously reported ovarian cancer-specific IKKε signature genes enriched in cellular invasion and metastasis function, and a modest decrease in cellular growth upon the loss of IKKε [5]. To identify genes whose depletion further inhibit the proliferation and survival of OC cells in combination with IKKε depletion, we first created IKKε matched pseudo-isogenic cell lines by stable knockdown of IKKε or control shRNA followed by magnetic beads purification. When introducing the shRNA library in quadruplicate (Figure 1A, Supplementary figure 1), we focused on kinome targets from a barcode-tagged shRNA library [8], with the goal of identifying a molecule that compounded growth inhibition when knocked down in combination with IKKε, and would be amenable to chemical inhibition in subsequent studies. The knockdown of IKKε was well-maintained for up to 12 doublings without significant loss of purity, and the purity of shRNA library was similarly efficient in the magnetic beads purified control and IKKε-depleted cells (Figure 1B–D). Significant differences between shRNAs remaining at given time points were identified by sequencing barcode tags in IKKε-depleted cells compared to control. In order to prioritize candidate targets, we identified shRNAs depleted at two different time points (Figure 2A). Sixty-five genes were identified with a fold difference less than 0.7 and *p*-value less than 0.05 when comparing IKKε-depleted cells to control (Supplementary table 1). These 65 genes were most significantly involved in cellular proliferation/growth, cancer, and cellular death/survival pathways based on Ingenuity Pathway Analysis (IPA) (Figure 2B). Accordingly, many genes were networked with p38 MAPK, PI3K, and NF-κB complexes (Figure 2C). In order to prioritize clinical relevance to OC, we examined the expression levels of these 65 genes in The Cancer Genome Atlas (TCGA) containing more than 500 ovarian serous cystadenocarcinoma [2]. Three genes - *CHEK1*, *EPHB3*, and *PIP5K1A* - were increased at least 2 fold in expression in more than 50% of the tumor set compared to non-cancer controls. Strikingly, CHEK1 was overexpressed by 1.5-fold in 100% of TCGA OCs. The frequent over expression of CHEK1 in clinical specimens suggests that it is relevant to the biology of OC. Therefore, we focused further investigation on CHEK1.

### The lethality of CHEK1 loss in IKKε-depleted OC cells is validated

**Figure 3:**
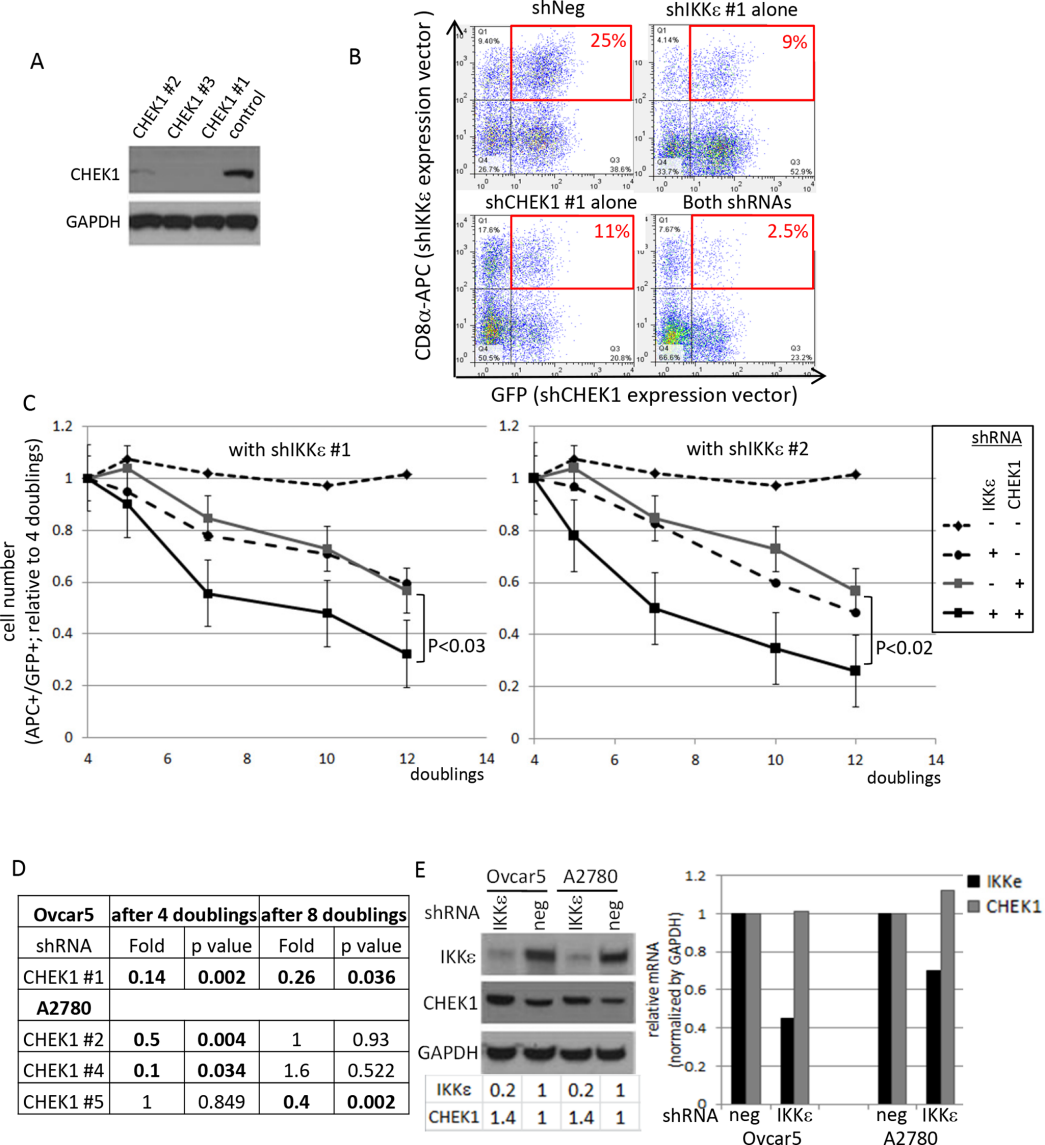
Validation of CHEK1 lethal effects in IKKε-mtached Ovcar5 cells **(A)** The knockdown levels of CHEK1 by 3 different shRNAs were examined by Western blot analysis. GAPDH was used as a loading control. **(B)** Double-positive cells after IKKε and CHEK1 shRNA knockdown were monitored by flow cytometry after two rounds of infection. Dot plots at 7 doubling times were shown. IKKε shRNA constructs co-express LYT2 (mouse CD8α) which was detected by an antibody conjugated to APC; CHEK1 shRNA constructs co-expressed GFP. **(C)** Two previously validated IKKε shRNAs were used. Either IKKε #1 or IKKε #2 shRNA (in pRSM-LYT2 vector) was co-infected with one of 3 different CHEK1 shRNAs (in pRSMX-PG vector) into Ovcar5 cells. The changes in double-positive cells were measured and calculated relative to 4 doublings after infection, at which GFP signal is maximal. The average number from 3 different CHEK1 shRNA in either negative shRNA or IKKε shRNA were calculated during indicated time courses. The significant *p* value was calculated by a 2-sided student's t-test. **(D)** A2780 IKKε-matched cell lines were established and shRNA library screening performed in the same manner as in Ovcar5. Using a cut-off with a fold change of ≤ 0.7 with a *p*-value of ≤ 0.05 shown in bold, 3 different CHEK1 shRNAs were identified at the different time points. **(E)** The expression levels of IKKε and CHEK1 proteins (left) and mRNAs (right) were examined upon IKKε knockdown in two cell lines. GAPDH was used as a loading control for immune blotting. The area of each band was quantified by ImageJ and normalized by GAPDH, and expressed as the level relative to its negative control. The mRNA levels were quantified by real-time PCR, normalized by that of GAPDH, and the expression was calculated relative to that in negative shRNA infected cells.

To validate the lethal effect of CHEK1 loss in the setting of diminished IKKε expression, we utilized 3 different CHEK1 shRNAs with two previously validated IKKε shRNAs [5]. The knockdown of CHEK1 was confirmed (Figure 3A). Either IKKε or control shRNA was co-infected with either CHEK1 or control shRNA, and dually positive cells were monitored (Figure 3B). IKKε knockdown alone showed a moderate effect on cellular survival as we reported previously [5]. Similarly, CHEK1 knockdown alone showed the same degree of decrease in survival as IKKε knockdown (Figure 3C). Consistent with the shRNA library screen, we observed a significant greater decrease in the number of the cells with co-knockdown of CHEK1 and IKKε compared to single knockdown of either IKKε or CHEK1 alone (Figure 3C). Additionally, we examined an independent screen in IKKε-matched A2780 cell lines, containing wild-type p53 to see whether the co-dependent lethality of IKKε and CHEK1 is p53-dependent. CHEK1 loss also decreased viability in conjunction with IKKε depletion in this p53 wild-type background, suggesting that this combined lethal effect is p53-independent (Figure 3D). Interestingly, the individual CHEK1 shRNAs identified in A2780 differed from those found significant in Ovcar5, perhaps implying that cellular kinetics for shRNA processing was different between the two cell lines. Nonetheless, three different CHEK1 shRNAs were selected and used for further validation in subsequent experiments. Of note, CHEK1 protein expression was slightly increased in IKKε-depleted Ovcar5 and A2780, while no significant changes in *CHEK1* mRNA levels were detected upon IKKε depletion (Figure 3E). This suggests that IKKε does not directly affect the transcriptional level of *CHEK1*.

Taken together, dual shRNA-mediated screening efficiently identified the co-dependence of CHEK1 and IKKε for OC proliferation and survival.

### Combined pharmacological inhibition of IKKε and CHEK1 is more potent than single treatment

Chemical inhibitors are more useful as therapeutic interventions, although they are less specific than RNAi and are unable to interrogate kinase-independent functions. Nonetheless, we investigated whether chemical inhibitors could recapitulate the effects of shRNAs. BX795 inhibits IKKε kinase activity. *In vitro*, BX795 can also inhibit TBK1, PDK1, Aurora B, MARK1, MARK2, MARK4, NUAK1, MLK1, MLK2, MLK3 with the range of 5–111 nM [9]. Of note, MARK2 was also identified as IKKε-dependent lethal gene (Supplementary table 1), but only 4 out of 506 TCGA ovarian tumors showed overexpression (2 fold change) of MARK2, which functions in cell polarity and regulation of microtubule dynamics. PF477736 inhibits CHEK1 at picomolar concentration (490 pM) and it also affects CHEK2, VEGFR2, YES and FMS in the range of 8–47 nM in non-cellular systems [10].

**Figure 4:**
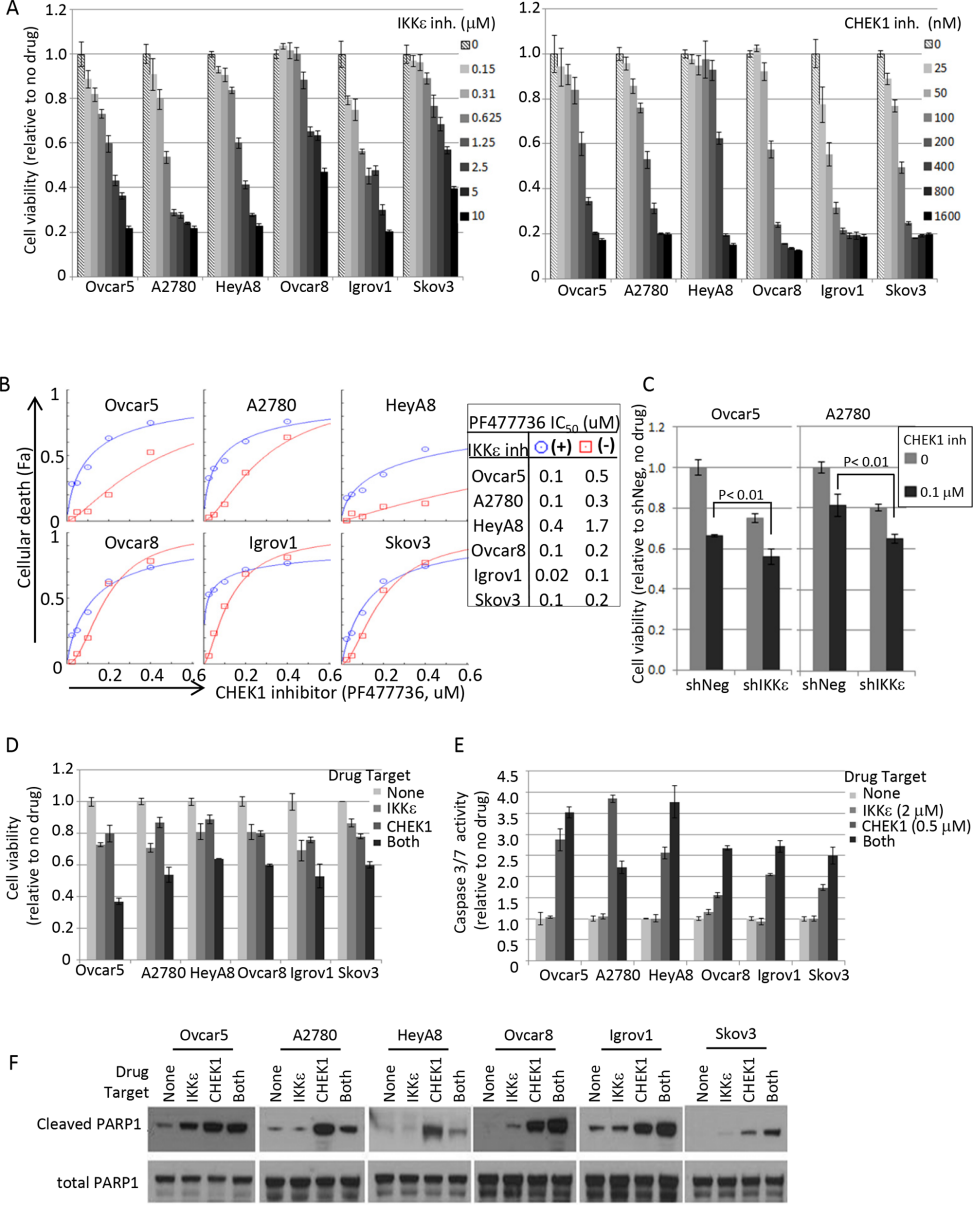
Pharmacological intervention of IKKε and CHEK1 in ovarian cancer cell lines **(A)** IKKε inhibitor (BX795) and CHEK1 inhibitor (PF477736) sensitivities were measured by XTT assay. Cells were seeded at 1000 cells/well in 50 μl and 4 replicates 16-20 hours prior to the addition of the drug. XTT assay was performed 3 days later upon drug treatment. The viability was calculated relative to no drug treated samples. **(B)** Cellular viability assay was performed in 2-fold serial dilutions of PF477736 (range 0 – 0.4 μM) in triplicate in a 96 well matrix format, and then cellular death (Fa; fraction affected) was calculated from XTT based viability fractions. IC50 was calculated using CompuSyn software. Shown are the results in the presence of BX795 (circle; 1.25 μM, except 0.625 μM for A2780) and the absence of the IKKε inhibitor (square). **(C)** IKKε and CHEK1 doubly depleted Ovcar5 and A2780 cells maintained in selective media (25 μg/ml of mycophenolic acid and 2 μg/ml of puromycin) were seeded in 96-well plates 24 hours prior to the addition of CHEK1 inhibitor PF477736. XTT assay was performed 3 days later upon drug treatment. Statistical tests were 2-sided between shNeg and shIKKε in the presence of CHEK1 inhibitor. **(D)** The relative cell viability was calculated based on no drug treatment, and is shown at the following suboptimal concentrations of BX795/PF477736: 1.25 μM/0.2 μM (Ovcar5, HeyA8), 1.25 μM/0.1 μM (Ovcar8, Skov3), 0.625 μM/0.1 μM (A2780), and 0.625 μM/0.05 μM (Igrov1). SD was calculated from 3 replicates. XTT assay was performed 3 days after treatment. **(E)** Caspase-3/7 activity was measured by Caspase-Glo assay after exposure to BX795 (2 μM) and/or PF477736 (0.5 μM) for 24 hours. Mean values ± SD of 3 replicates are shown and the data represent 4 independent experiments. **(F)** The cleavage of apoptotic indicator PARP-1 was examined by Western blot analysis. Cells were treated for 24 hours as the same conditions as in Figure 4E and harvested for total cell lysate preparation. Total PARP1 is shown as a loading control.

We asked whether the inhibition of IKKε and CHEK1 kinase functions was critical to the cooperative decrease in cellular viability detected by the initial shRNA-mediated screening methodology. The status of p53 -whether wild-type, mutant, or null- could affect anti-apoptotic and cell cycle functions of CHEK1. Thus, we included 6 OC cell lines expressing high levels of IKKε with different p53 status in this pharmacological investigation. We first examined the expression levels of IKKε, CHEK1, and p53 and confirmed the status of p53 mutation in each cell line by mRNA sequencing (Supplementary figure 2). A2780 was relatively sensitive to IKKε inhibitor BX795, with an IC_50_ of approximately 1 μM, while Ovcar8 and Skov3 were more resistant (Figure 4A-left). Interestingly, the sensitivity to CHEK1 inhibitor (PF477736) was independent of the p53 status and IC_50_ was in the range of 100–500 nM (Figure 4A-right).

Consistent with the shRNA results, the IC_50_ of CHEK1 inhibitor was consistently lower in the presence of IKKε inhibitor in all six cell lines (Figure 4B). When we tested the sensitivity of CHEK1 inhibitor in IKKε-depleted cells, we consistently observed that IKKε-depleted (shIKKε) cells were more sensitive to CHEK1 inhibitor than IKKε-expressed (shNeg) cells in both Ovcar5 and A2780 cells (Figure 4C). When the drug synergism of IKKε and CHEK1 inhibitors was determined by XTT assay after 3 day treatment, we found that each cell line required different optimal concentrations of IKKε and CHEK1 inhibitors to achieve the maximal synergistic or additive effect as shown in the combination index (CI) values (Supplementary figure 3). Because IKKε inhibitor was relatively cytostatic compared to CHEK1 inhibitor and showed linear cellular toxicity at the lower concentrations, CI indicated either additive or synergistic effects (CI ≤ 1) at the lower concentration range, but the effect generally became antagonistic (CI > 1) at the higher range in 6 ovarian cancer cell lines. Accordingly, when tested at concentrations where single agent exposure showed minimal effect, the co-inhibition of IKKε and CHEK1 significantly decreased cellular viability (Figure 4D). The decrease in cellular viability with the co-treatment was generally consistent with increased apoptosis, measured by caspase3/7 activity and cleaved PARP1, suggesting that inhibition of both IKKε and CHEK1 primed OC cells for apoptosis (Figure 4E–F). Importantly, the treatment with IKKε inhibitor alone did not induce caspase3/7 activity and produced no or minimal PARP cleavage, suggesting that blockage of IKKε kinase activity does not induce apoptosis. Overall, combined inhibition of IKKε and CHEK1, regardless of p53 status, was more effective in killing OC cells than single treatment.

### IKKε and CHEK1 inhibition have different effects on DNA damage response and cell cycle

**Figure 5:**
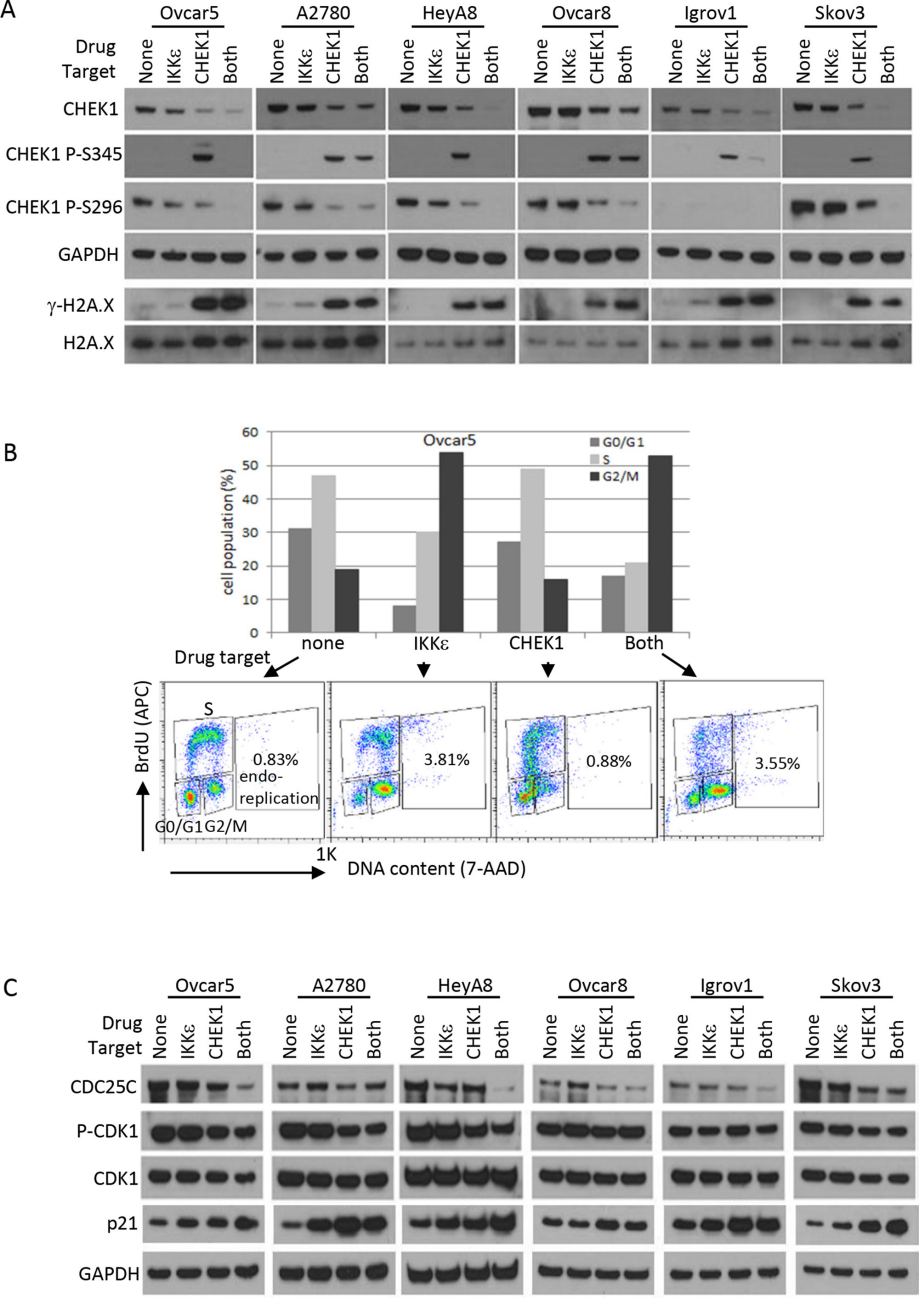
DNA damage response and cell cycle analysis after CHEK1 and IKKε inhibition **(A)** Western blotting for DNA damage response. Phosphorylated CHEK1, total CHEK1, and γ-H2A.X were measured by Western blotting after 24 hour treatment at final concentrations of 2 μM (BX795) and/or 0.5 μM (PF477736) prepared in fresh medium. GAPDH and H2A.X were used as loading controls. **(B)** Cell cycle was analyzed after pharmacological intervention in Ovcar5. Cells were treated for 16 hours at final concentrations of 2 μM (BX795) and/or 0.5 μM (PF477736) prepared in fresh medium. G_0_/G_1_, S, and G_2_/M phases were measured based on staining of APC-BrdU and 7-AAD by flow cytometry. **(C)** Western blotting for cell cycle regulators. The treatment conditions and total cellular lysates preparations are same as in Figure 4E–F.

CHEK1 is known to be involved in DNA damage response and cell cycle regulation. CHEK1 inhibitor increased CHEK1 phosphorylation at Ser345 and decreased total CHEK1 level, indicators of DNA damage response (Figure 5A). This likely indicates that cells were no longer able to repair endogenous levels of DNA damage when CHEK1 was inhibited. IKKε inhibition alone did not trigger CHEK1 S345 phosphorylation (Figure 5A). The c-terminal domain of CHEK1 contains regulatory phosphorylation sites (S296, S317, and S345) that inhibit the N-terminal catalytic domain through a proposed intramolecular interaction [11, 12]. Interestingly, CHEK1 S296 phosphorylation, an autophosphorylation followed by ATR-induced S317 and S345 phosphorylations upon DNA damage, was detected in the absence of CHEK1 S345 phosphorylation in all cell lines except Igrov1, a relatively lower CHEK1-expressing cell line (Supplementary figure 2). Therefore, it is tempting to speculate that CHEK1 is phosphorylated at S296 through a homodimer formation at higher levels of CHEK1 expression, such as that found in OC. This autophosphorylation would serve to maintain active CHEK1 in the nucleus. Nonetheless, the inhibition of both IKKε and CHEK1 further decreased total CHEK1 levels compared to single treatment. Consistent with no changes in CHEK1 S345 phosphorylation, no gamma-H2A.X was detected in cells treated IKKε inhibitor alone, indicating an insignificant amount of intrinsic DNA damage accumulated in these cells. As expected, gamma-H2A.X level was increased in cells exposed to CHEK1 inhibitor alone or combined with IKKε inhibitor. Therefore, while the inhibition of IKKε did not trigger DNA damage response and minimally induced apoptosis, the combined inhibition of both IKKε and CHEK1 induced DNA damage and apoptosis.

Next, we investigated the cell cycle effects of CHEK1 and IKKε. Interestingly, IKKε inhibitor alone consistently induced dramatic G2/M arrest; CHEK1 inhibitor alone caused abnormal S phase profile in p53 mutant and null cell lines, and G1 arrest in A2780, a p53 wild-type cell line (Figure 5B, Supplementary figure 4A). Co-treatment with both inhibitors showed a mixed profile, presenting an increase in G2/M population and abnormal S phase. Curiously, shRNA-mediated knockdown of either IKKε or CHEK1 alone showed an increase in G0/G1 population and in a decrease in S, and the dual knockdown of IKKε and CHEK1 showed further increases in the G0/G1 in both Ovcar5 and A2780 cells (Supplementary figure 4B). This discrepancy may be due to kinase independent functions of IKKε and CHEK1 and/or the perturbation of IKKε-interacting partners.

Interestingly, cells reproducibly showed an increase in endoreplication, aberrant DNA replication without proper cytokinesis, upon IKKε inhibition. This suggests that the kinase activity of IKKε may be involved in mitosis (Figure 5B, Supplementary figure 4A). We did not observe an increase in the level of phospho-H3 (ser10), an indicator of mitosis, in these G2/M arrested samples, implying that the arrest occurred mostly in the G2 phase, prior to cells entering mitosis (data not shown). We also measured proteins involved in a G2 to M transition to determine the effects of the combined inhibition. CDK1 is phosphorylated in the G2 phase and then dephosphorylated by CDC25C to promote entry into M phase. CHEK1 inhibits CDC25C activity upon DNA damage to trigger G2 arrest, and allow cells to repair DNA prior to entering mitosis. We therefore hypothesized that IKKε may compound the effect of CHEK1 on these proteins, as a molecular explanation for our observed effects on cell cycle. Consistent with this hypothesis, CDC25C decreased in all OC cell lines treated with CHEK1 inhibitor, and further decreased with combined inhibition of IKKε and CHEK1 (Figure 5C). Phospho-CDK1 and total CDK1 levels were also lowered. The p21 protein is known to induce G2 arrest [13, 14], and we observed that inhibition of IKKε and/or CHEK1 increased p21 protein level in OC cells (Figure 5C). In summary, inhibition of IKKε kinase activity alone resulted in G2 arrest without causing DNA damage response, while the combination of IKKε and CHEK1 inhibition showed both DNA damage and G2 arrest, with an increase in apoptosis.

### IKKε suppresses p21 protein level in its kinase dependent manner to block apoptosis

**Figure 6:**
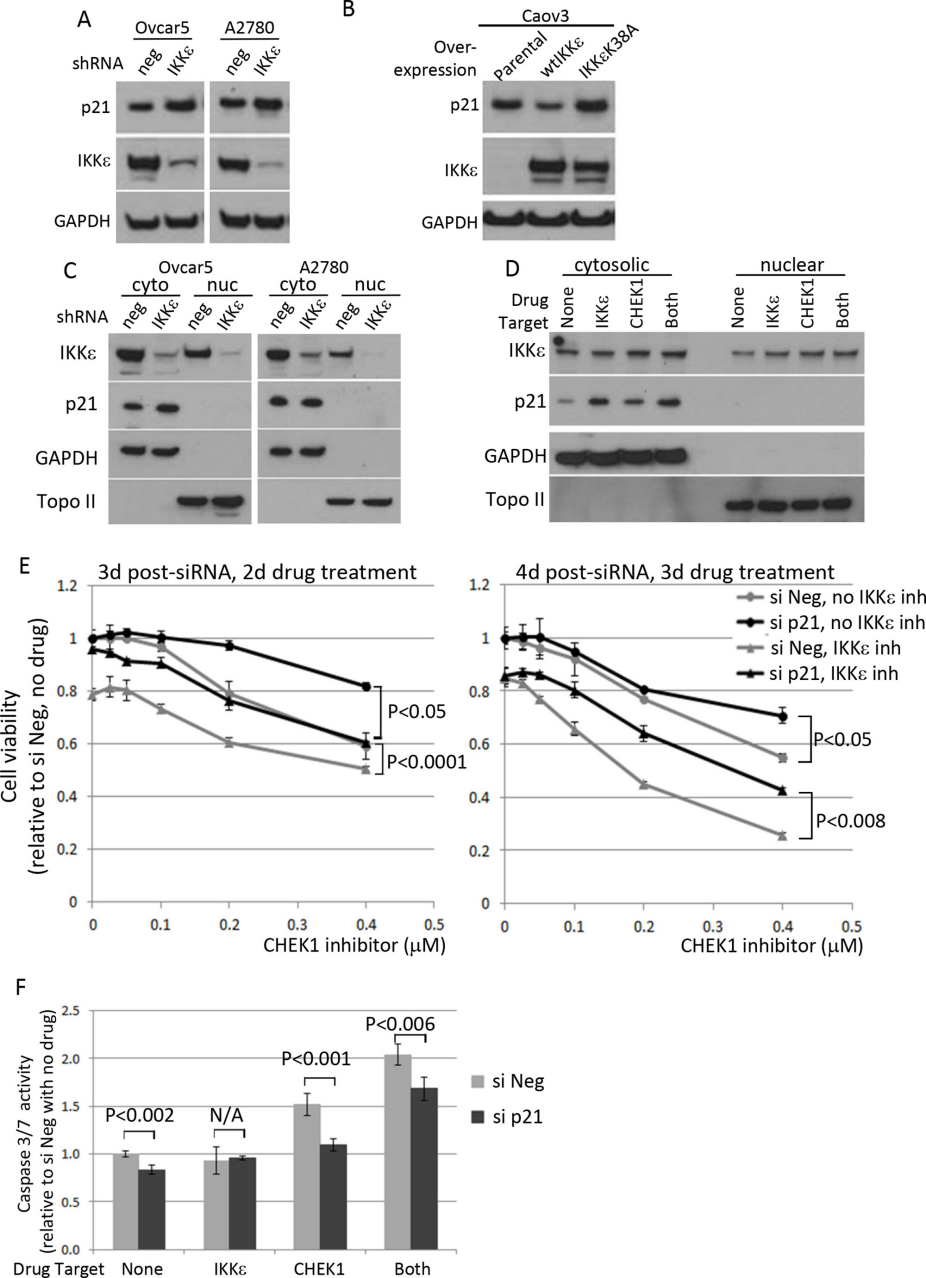
The effect of IKKε manipulation on p21 level and its effect on survival of ovarian cancer cells **(A)** Ovcar5 and A2780 were depleted of IKKε or negative control and maintained under selection (25 μg/ml of mycophenolic acid). Total protein lysates were prepared to examine p21 level and the knockdown of IKKε. GAPDH was used as a loading control. **(B)** Caov3-wtIKKε and Caov3-IKKε K38A were maintained in selective media (100 μg/ml of G418) and total protein lysates were prepared for the protein analysis. **(C)** Cytoplasimc and nuclear protein fractionation was prepared using Ovcar5 and A2780 depleted for IKKε (shRNA#1) or control and maintained in culture after magnetic beads purification for LYT2 surface selection marker. **(D)** Ovcar5 cells were treated for 4 hours with IKKε inhibitor (BX795, 2 μM) and/or CHEK1 inhibitor (PF477736, 0.5 μM) prepared in fresh medium prior to cellular fractionation. GAPDH and Topoisomerase II were used as cytosolic and nuclear markers, respectively. **(E)** Cells transfected with either siNeg or si p21 were seeded at 2000 cells/well in 50 μl in 3 replicates and 8 hours later PF477736 was added in the absence or presence of BX795 (0.3 μM). XTT assay was performed 2 and 3 days later upon drug treatment. The viability was calculated relative to no drug and siNeg treated samples. The significant *p* values between siNeg and si p21 samples were calculated by a 2-sided student's t-test. **(F)** After 16 hours siRNA transfection, cells were seeded and treated for 16 hours with IKKε inhibitor (BX795, 0.3 μM) and/or CHEK1 inhibitor (PF477736, 0.4 μM) in 4 replicates. The caspase3/7 activities were shown relative to untreated siNeg samples from 4 replicates. The significant *p* values between siNeg and si p21 samples were calculated by a 2-sided student's t-test.

Most of p21 and IKKε were expressed in cytosolic fraction of OC cell lines (Supplementary figure 5A). Since the inhibition of IKKε kinase activity resulted in an increase in p21 level in Ovcar5 and A2780 cells, we measured changes in p21 level upon loss- and gain- of IKKε expression. In both cell lines, p21 level increased when IKKε was depleted (Figure 6A). Complementary to the knockdown experiments, p21 level was decreased, when wild-type IKKε was overexpressed in OC cell line Caov3, that expresses no and very low endogenous levels of IKKε and CHEK1, respectively; the overexpression of a kinase dead mutant IKKε K38A did not affect p21 level (Figure 6B). However, neither manipulation of IKKε level nor its kinase inhibition affected the cytosolic localization of p21 (Figure 6C–D).

Since either inhibition or depletion of IKKε and CHEK1 resulted in an increase in p21 level and apoptosis, we hypothesized that p21 was required for inducing cell death under these conditions. Ovcar5 cells were transiently depleted for p21 by siRNA and then treated with a range of CHEK1 inhibitor in the absence and presence of IKKε inhibitor. Of note, the knockdown of p21 was confirmed and did not affect the levels of IKKε and CHEK1 (Supplementary figure 5B). As predicted, the cells depleted of p21 were more resistant to CHEK1 inhibitor both in the absence and presence of IKKε inhibitor compared to the cells treated with siNeg control after 2 and 3 days drug treatment (Figure 6E). Accordingly, apoptosis was decreased in p21 siRNA samples compared with siNeg controls (Figure 6F). This suggests that OC cell death with the combined treatment was in part mediated by p21. Taken together, these results imply that IKKε promotes OC survival by maintaining a low level of p21, and that p21 increases cell death upon the combined inhibition of IKKε and CHEK1 in OC cells.

## DISCUSSION

In this study, rapidly generated shRNA-mediated pseudo-isogenic cell lines were an efficient tool to discover signaling interactions on a genomic level. We discovered that the loss of CHEK1 sensitizes IKKε-depleted OC cells. Translation to co-treatment with chemical inhibitors of CHEK1 and IKKε resulted in the cooperative decrease in cellular viability and a further increase in apoptosis. Therefore, our finding of IKKε and CHEK1 interplay may lead to development of context-specific therapeutics in the poor prognostic group of patients with a high level of IKKε expression. Additionally, the expression level of IKKε may assist in directing therapy to a cohort of patients most likely to respond to targeted intervention. As such, we are currently exploring IKKε as a potential marker of resistance in a clinical trial of CHEK1 inhibitor, given our current findings suggesting that IKKε acts as a compensatory pro-survival and anti-apoptotic molecule in OC.

Synthetic lethality screening is a useful tool to identify gene-to-gene interactions and has been applied to the development of cancer- or context-specific cytotoxic agents. Here, we quickly established IKKε pseudo-isogenic cell lines by transduction of IKKε or control shRNA and subsequent enrichment by magnetic bead purification. IKKε sensitization screening identified 65 candidate genes; of these, *CHEK1*, *EPHB3*, and *PIP5K1A* are overexpressed in a majority of OC. Overexpression of Eph receptors and ephrin ligands are reported in human tumors and associated with poor prognosis, although the signaling is characterized by complex dichotonomies (reviewed in [15]). *PIP5K1A* encodes phosphatidylinositol phosphate 5-kinase α protein, which synthesizes PIP_2_. PIP5K1A is shown to be a caspase-3 substrate and its overexpression suppresses apoptosis [16]. We set a lower priority for EPHB3 and PIP5K1A because of the lack of ability to move forward with tool compounds and pharmacologic inhibitors.

We chose to focus on CHEK1 due to its clinical applicability, and relevance to OC. CHEK1 is a central regulator of the cellular response upon DNA replication block and DNA damage [17, 18]. CHEK1 has been an attractive target for cancer treatment, especially for p53-deficient cancers such as OC. In response to DNA damage by chemotherapy and radiotherapy, CHEK1 arrests p53-deficient cancers in the G2 phase by coordinating various aspects of DNA repair to avoid mitotic catastrophe. Therefore, CHEK1 inhibitors have been developed as anti-cancer agents (reviewed in [19]). Unfortunately, very few clinical responses were achieved in patients with solid tumors treated with single-agent CHEK1 inhibitors or when used in combination with chemotherapy [20, 21]. Such disappointing clinical results may be due to compensatory signaling pathways present in the cancer cells. This novel interplay between IKKε and CHEK1 described here provides one possible explanation as to why very few clinical responses were achieved in patients with solid tumors treated with single-agent CHEK1 inhibitors. IKKε activity may block apoptosis and perpetuate the cell cycle in CHEK1-inhibited cells, thus allowing cancer cells to survive and proliferate with compromised DNA.

**Figure 7:**
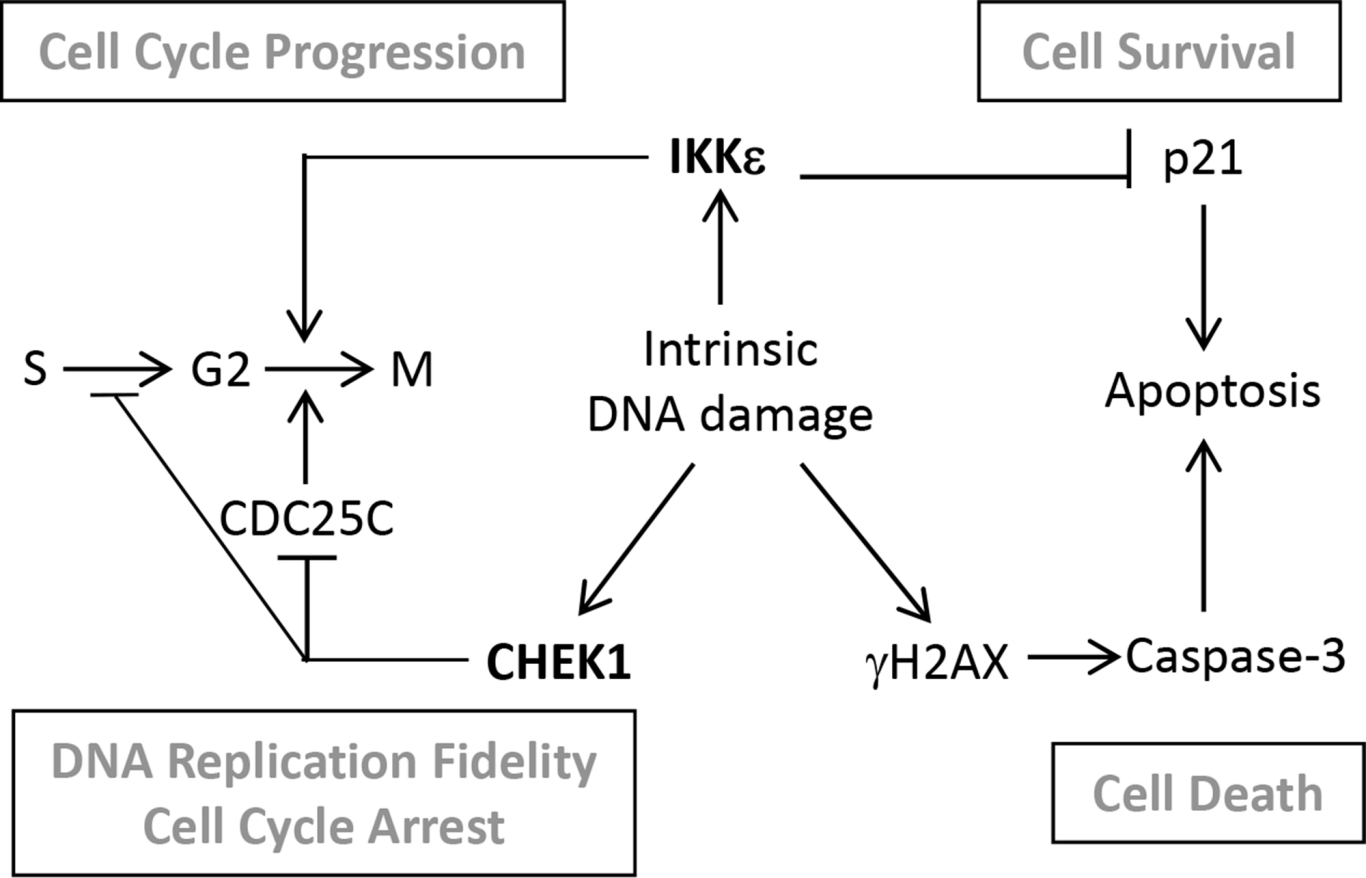
Schematic diagram of a proposed interplay between CHEK1 and IKKε in OC cells In ovarian cancer cells characterized with high genomic instability, intrinsic DNA damage is repaired by overexpressed CHEK1. Through a pathological balance between IKKε and CHEK1, G2 to M transition is either promoted or blocked depending on the level of intrinsic DNA damage to facilitate cellular proliferation and survival. Ovarian cancer cells expressing high levels of IKKε block apoptosis by maintaining the low level of p21 to support cellular survival. When IKKε and CHEK1 are inhibited, accumulated intrinsic DNA damage and increased level of p21 trigger cellular apoptosis.

IKKε was identified as an oncogene amplified in breast cancer [3]. We previously demonstrated IKKε as a coordinator in OC metastasis and invasion by modulating IKKε expression levels in both loss- and gain-of-function systems [5]. A recent study described a protective role of IKKε in DNA damage induced apoptosis through its kinase-dependent nuclear translocation [22]. Our data presented here suggest a model in which IKKε and CHEK1 cooperate to promote OC cell viability by the anti-apoptotic and pro-survival function of IKKε upon DNA damage, and DNA damage repair triggered by CHEK1 (Figure 7). In support of this model, pro-survival proteins such as CDK1 and CDC25C, as well as DNA damage checkpoint protein CHEK1 were dramatically decreased upon co-inhibition of both IKKε and CHEK1. In addition, the kinase function of IKKε contributes to cell cycle regulation. Interestingly, p21 protein expression was mainly detected in the cytosolic compartment of OC cell lines at the steady state level, increased with inhibition or loss of IKKε, and decreased with overexpression of wild-type IKKε. The increase of p21 protein coincided with increased apoptosis in dually inhibited cells. Curiously, the treatment with IKKε inhibitor alone also elevated p21 level, but there were no changes in apoptosis and DNA damage markers. Even now, the exact role of p21 in cancer is not clear, as it can exhibit both tumor suppressive and promoting activities as well as pro-apoptotic and anti-apoptotic functions [14]. For example, taxol-induced p21 up-regulation or overexpression of p21 in breast cancer cells induced growth arrest and apoptosis [23, 24]. In addition, ectopic expression of p21 in OC cell lines led to increased cisplatin-induced apoptosis [25]. Paradoxically, increased cytoplasmic p21 expression in OC cell line C13* was correlated with cisplatin resistance and knockdown of p21 by siRNA rendered the cells sensitive to cisplatin by increased apoptosis [26]. Our current study implicates an apoptotic role of p21 upon co-inhibition of IKKε and CHEK1 in OC cells.

In summary, we have developed a fast and efficient dual shRNA technique to uncover synthetic lethal combinations, obviating the need to establish conventional isogenic cell line models or to develop potent and specific tool compounds. Due to its rapid and simple procedure, this can be performed simultaneously in multiple cell lines within a few weeks of time frame. With this technique, we efficiently identified CHEK1 as an IKKε-dependent lethal gene, and identified a mechanism by which these two proteins balance cell cycle regulation, DNA repair and apoptosis. Many OCs have an underlying defect in DNA repair, leading to genomic instability and initial sensitivity to DNA damaging therapies, which subsequently fail due to tumor evolution. Our findings describe interplay between IKKε and CHEK1 in controlling cell cycle and DNA repair pathways, and direct to a novel point of intervention to focus clinical development for OC treatment. In addition, identification of interdependence between CHEK1 and IKKε in OC led us to test IKKε as an exploratory marker for resistance to CHEK1 inhibitors in the clinic.

## METHODS

### Cell lines

The source and authentication of ovarian cancer cell lines, A2780, Ovcar5, Ovcar8, HeyA8, Igrov1, and Skov3, were previously described [27] and maintained in RPMI supplemented with 10% heat-inactivated FBS and 1X Pen/Strep. CeB cells used for virus production were maintained in DMEM supplemented with 10% heat-inactivated FBS and 1X Pen/Strep. Caov3-IKKε K38A cell line was established as previously described for Caov3-wtIKKε cell line [5].

### Plasmids

IKKε shRNAs and negative control E_1/2_ shRNA were cloned into a pRSMX-LYT2 [28] vector using BsmI and XbaI sites. Plasmid pBabe-Neo-Flag-IKBKE K38A was generated by QuikChange II XL Site-Directed Mutagenesis Kit (Agilent) using a wild-type pBabe-Neo-Flag-IKBKE and the following primers;

Forward-GGAGAGCTGGTTGCTGTG*GC*GGTCTTCAACACTACCAGC
Reverse-GCTGGTAGTGTTGAAGACC*GC*CACAGCAACCAGCTCTCC

### Establishment of IKKε-matched cell line

Either IKKε #1 shRNA (previously named as B6 [5]) or negative E_1/2_ shRNA (IKKε shRNA without the complementary hairpin sequences) in a pRSMX-LYT2 plasmid was co-transfected with helper plasmids pHIT456 and pHIT60 into a CeB packaging cell line using Lipofectamine 2000. Two rounds of viral supernatants were then applied to OC cell lines over the course of 48h. After one doubling time, cells were detached using Cellstripper (Mediatech, Inc. Manassas, VA). Twenty million cells were resuspended in 320 μl of I buffer (1 × PBS containing 5% BSA and 2 mM EDTA), and then 80 μl of CD8α microbeads (Miltenyl Biotec) were added. After incubating on ice for 20 minutes, cells were washed with 8 ml of I buffer. Five to ten million cells in 500 μl of I buffer were loaded on the MACS MS separation column which was installed on the magnetic separator. After washing 3 times with 500 μl of I buffer, the column was removed from the separator and the CD8α-labelled cells were flushed out with 1 ml of complete medium and recovered for 2 days before next experiments.

### Protein kinase shRNA library screening

Five shRNA pools (about 6400 shRNAs) [8] targeting total 521 protein kinase genes were separately packaged in 6 well plates and split in half to infect IKKε-matched cell lines. After two rounds of infection in 4 biological replicates, the library-infected A2780 and Ovcar5 cells were selected at a final concentration of 2 μg/ml of puromycin for 2 and 4 days, respectively. The cells were harvested at the indicated time points after completing puromycin selection. To sequence the bar code from the library infected cells, genomic DNAs were isolated using DNeasy blood & tissue kit (Qiagen). The bar codes were amplified using TaKaRa LA Taq hot start polymerase (Takara Bio Inc.) with universal forward primer and 8 different reverse primers as shown in supplementary table 2. PCR reaction was performed in a 100 μl volume containing 0.9 μg of gDNA, 0.5 μM of forward and reverse primers, 5 U of TaKaRa LA Taq, 1X LA buffer, and 16 μl of dNTP mixture (2.5 mM each), following the PCR conditions as recommended by manufacturer. Each PCR product was checked on the 1.5% agarose gel and cleaned by MiniElute PCR purification kit (Qiagen). At this point, 1μg of each PCR product from samples No 1-8 in A2780 days 0, 4, 7 and Ovcar5 days 0, 7, 14 was combined and further purified by Agencourt AMPure XP system (Beckman Coulter) and eluted with 40 μl of ddH_2_O. Thirty microliters of 1:10 diluted samples were sent for sequencing (NCI, CCR-sequencing facility). The sequencing was done by Illumina GA IIx with TrueSeq 2.0, alignment was done by Illumina Casava 1.8.1, and HG19 was used as a reference genome.

### shRNA library analysis

An over-dispersed Poisson distribution was used to model the distribution of bar code reads for a given shRNA in a given experiment, and the statistical significance of observed differences between experiments was calculated. Full details can be found in the supplementary method 1. After bar codes were identified and matched to target genes, candidate targets were identified using a cut-off with a fold change of ≤ 0.7 with a *p*-value of ≤ 0.05 in the comparison between IKKε-depleted cells and its negative control counterpart. The sequences of *IKK*ε and *CHEK1* shRNAs used in validation experiments are in Supplementary Information.

### Establishment of stable double-knockdown of IKKε and CHEK1 cells

IKKε shRNAs and negative control E_1/2_ shRNA were cloned into a pRSMX-IMPDH vector using XhoI and BstBI sites in order to allow double selection using mycophenolic acid and puromycin. Virus supernatants of either IKKε or negative control shRNA in pRSMX-IMPDH and either negative control or CHEK1 shRNA in pRSMX-PG were mixed at a ratio of 1:1 and applied to cells twice over the course of 48h. Cells were selected for 7 days, at which no survived cells were observed in uninfected control plates, in the presence of 25 μg/ml of mycophenolic acid and 2 μg/ml of puromycin.

### Western blot analysis

Total protein was extracted from OC cell lines with 1% NP40 lysis buffer containing 150 mM NaCl, 50 mM TrisHCl, 10% glycerol, 1 × Halt proteinase inhibitor cocktail, 5 mM NaF, and 1 mM NaOrthovanadate. Protein concentrations were estimated using BCA Protein Assay Kit (Thermo Scientific, Rockford, IL). The proteins were separated on the NuPage 4–12% gel (Invitrogen, Carlsbad, CA) and the band was visualized using either Lumina Classico or Crescendo Western HRP substrate system (Millipore) depending on the signal intensities. Antibodies IKKε (Sigma, I4907), CHK1 G-4 (Santa Cruz, sc-8408), phospho-CHK1 Ser345 (Cell Signaling, #2348), phospho-CHK1 Ser296 (Cell Signaling, #2349), PARP-1 (Santa Cruz, sc-7150), cleaved PARP (Cell Signaling, #9541), phospho-H2A.X (Cell Signaling, #5438), H2A.X (Abcam, ab10475), Phospho-cdc2 (Tyr15) (Cell Signaling, #9111), cdc2 (Cell Signaling, #9112), p21 (Millipore, #05-345), p53-BP53.122 (Santa Cruz, sc-73566), and GAPDH (Millipore, MAB374), Topoisomerase II (Abcam, ab52934) were used in this study, and the secondary antibodies ECL anti-rabbit IgG HRP and ECL anti-mouse IgG HRP (GE Healthcare) were used at 1:5000 dilutions.

### Quantitative PCR

Total RNA was isolated using RNeasy Mini kit (Qiagen, 74104) with on-column DNAse treatment, per manufacturer's instructions and detailed in Supplementary Information.

### XTT assay

Cells were seeded in 96-well plates at a density of 1-2,000 cells/50 μl/well. In general, the drug was added 24 hours after seeding and XTT assay was performed in 3 days after drug treatment. Cellular viablity was assessed by incubating cultures with XTT freshly mixed with PMS (Sigma) and absorbances were read in a Tecan plate reader (Research Triangle Park, NC). Cellular proliferation was calculated relative to experimental negative controls.

### Caspase3/7 assay

Cells were seeded in 96-well white-walled plates at a density of 5,000 cells/50 μl/well and the drug in a 50 μl volume was added 24 hours after seeding. The caspase activity was measured after 24 hours additional incubation followed by adding 40 μl Caspase-Glo reagent (Promega) per well.

### Cell cycle analysis

For cell cycle analysis of shRNA infected cells, cells were seeded at 1 × 10^6^/60 mm plate, 24 hours prior to addition of BrdU. The procedure was performed according to manufacturer's protocol. For cell cycle analysis of drug treated cells, 4-5 × 10^5^ cells/60 mm plate were seeded 24 hours prior to addition of drug. Fresh complete medium containing 2 μM BX795, 0.5 μM PF000477736, or both drugs was added and cells were incubated for 16 hours. Following 30 minutes incubation after adding BrudU, cells were harvested for subsequent fixation and permeabilization. Cells were analyzed by FACS Calibur (Becton Dickinson) after staining with APC-anti-BrdU and 7-AAD. Cell cycle was analyzed using FlowJo software.

### p21 knockdown

SMARTpool ON-TARGET plus human CDKN1A (Cat.# L-003471-00) and negative siRNAs (Cat. # D-001810-10) were purchased from Thermo Scientific. siRNAs were transiently transfected using DharmaFECT1 at a final concentration of 20 nM 16 hours prior to seeding onto 96-well plates at a density of 2000 cells per well. After 8 hours allowing cells to attach onto the plate, drugs were added. XTT assay were performed at 2 and 3 days after adding drugs. For capase3/7 activity assay with p21 knockdown experiments, 5000 cells were plated at 16 hours post-transfection, incubated for 6 hours, and treated with indicated drugs for 16 hours before measuring caspase3/7 activities.

### Cellular fractionation

Cytosolic and nuclear extracts were prepared by the Nuclear/cytosol fractionation kit (BioVision) according to the manufacturer's protocol.

## SUPPLEMENTARY METHODS FIGURES AND TABLES


