# Supplementary material for: Loss of compensatory pro-survival and anti-apoptotic modulator, IKKε, sensitizes ovarian cancer cells to CHEK1 loss through an increased level of p21
Source: Oncotarget. 2014 Nov 15;5(24):12788–802. doi: 10.18632/oncotarget.2665 (PMC4350339; doi:10.18632/oncotarget.2665)
Supplement: Supplementary file 1 [file oncotarget-05-12788-s001.pdf]

SUPPLEMENTARY METHODS

The sequences of *IKKε* and *CHEK1* shRNAs used in validation experiments:

IKKε #1 (B6)(Hsu and Kim et al 2012): GAGAAGTTCGTCTCGGTCTAT

IKKε #2 (E1)(Hsu and Kim et al 2012): GAGGAGTGCGTGCAGAAGTAT

CHEK1 #1: GAAGGCAATATCCAATATTTA

CHEK1 #2: GCTGCTATGTTGACATTATTC

CHEK1 #3: GCTCAGAGATTCTTCCATCAA

shRNA library analysis algorithm

Estimation when both experiments have at least one hit in one replicate

We assume that the number of counts on a given shRNA *i* of a given replicate *j* is distributed according to Poisson distribution with mean itself a random variable coming from a Gamma distribution. This results in the negative binomial probability distribution (Lawless 1987).

$$P(N_{ij} = k|a_i, \mu_{ij}) = \frac{\Gamma(k + a_i^{-1})}{k!\Gamma(a_i^{-1})}\left(\frac{a_i\mu_{ij}}{1 + a_i\mu_{ij}}\right)^k\left(\frac{1}{1 + a_i\mu_{ij}}\right)^{a_i^{-1}} \quad (1)$$

Where $a_i > 0$ represents the degree of technical variability between the replicates of a given shRNA, and μ_{ij} represents the expected mean.

We parameterize μ_{ij} as

$$\mu_{ij} \equiv T_{ij}e^{s_i}$$

where T_{ij} is a normalization factor that represents the loading of a particular shRNA pool in a given replicate, and s_i represents the relative biological representation of the given shRNA on a log scale.

To estimate T_{ij} we begin by defining a set of well expressed shRNAs

$$S \equiv \{i\text{:median}(N_{ij}) > 10\}$$

Then for each shRNA in a given pool P, we fix.

$$(T_{ij}|i \in P) = \text{mean}_{i \in S \text{ and } i \in P}(N_{ij})$$

We propose estimating s_i via maximum likelihood, such that $\mu_{ij} \equiv T_{ij}s_i$ satisfies

$$\sum_j \frac{N_{ij} - \mu_{ij}}{1 + a_i\mu_{ij}} = 0,$$

and estimating a_i by methods of moments such that

$$\sum_j \frac{(N_{ij} - \mu_{ij})^2}{\mu_{ij}(1 + a_i\mu_{ij})} = n - 1$$

where n is the number of replicates.

In practice solve these equations by performing alternating Newton Rhaphson approximations. Starting with setting

$$\hat{s}_i^0 = \log\left(\frac{\sum_j N_{ij}}{\sum_j T_{ij}}\right), \qquad \hat{a}_i^0 = 0$$

then iteratively defining

$$\hat{a}_i^{k+1} = \hat{a}_i^k - \frac{\sum_j\left(\frac{(N_{ij} - \hat{\mu}_{ij}^k)^2}{\hat{\mu}_{ij}^k(1 + a_i\hat{\mu}_{ij}^k)}\right) - n + 1}{\sum_j\left(\frac{N_{ij} - \hat{\mu}_{ij}^k}{1 + \hat{a}_i^k\hat{\mu}_{ij}^k}\right)^2}$$

and

$$s_i^{k+1} = s_{ij}^k + \frac{\sum_j\left(\frac{(N_{ij} - \hat{\mu}_{ij}^k)}{(1 + \hat{a}_i^k\hat{\mu}_{ij}^k)}\right)}{\sum_j\left(\frac{(1 + \hat{a}_i^k\hat{\mu}_{ij}^k)\mu_{ij}^k + (N_{ij} - \hat{\mu}_{ij}^k)\hat{a}_i^k\hat{\mu}_{ij}^k}{(1 + \hat{a}_i^k\hat{\mu}_{ij}^k)^2}\right)}$$

Where $\hat{\mu}_{ij}^k = T_{ij}\exp(\hat{s}_i^k)$. The exception to this rule is if a_i^{k+1} is negative we set $a_i^{k+1} = a_i^{k+1}/10$. We continue this iteration until successive estimates for s_i^k and a_i^k are within 0.001 of each other, or 500 iterations has elapsed. In the later case we flag the estimate as "Bad" and ignore it. Since we use a maximum likelihood estimate for s_i it should asymptotically be normally distributed with mean equal to the true s_i and variance error given by

$$\text{Var}(\hat{s}_\text{i}) \approx \sum_j \frac{\hat{\mu}_{ij}}{1 - \hat{a}_i\hat{\mu}_{ij}} \quad (2)$$

So then give two experiments if we take the difference between their estimates for s_i this will be the log ratio for the difference in presentation of the shRNA between the two experiments, and it should be normally distributed with variance equal to the sum of the variances of the individual estimates. From this we can derive *p*-values and Confidence intervals for this log ratio. Then the exponential of the confidence intervals becomes the confidence interval for the ratio.

The reported relative mean is

$$\text{Realative mean} = \text{median}_\text{j}(\text{T}_\text{ij})\exp(\hat{s}_i),$$

where the median is taken over all replicates in all experiments in the study.

Estimation when one of the experiments has 0 counts

The above analysis breaks down when one of the experiments has no counts in any of the replicates for a

given experiment. If both experiments have 0 counts, then there is clearly no significant effect and we set the *p*-value to 1. However in the case that one of the experiments has positive number of counts and the other has zero counts in all replicates we need to estimate the significance and magnitude of this effect since this may indicate a strongly active RNAI.

For simplicity of exposition let us assume the that first experiment resulted in a model with estimated parameters $\hat{s}_i$ and $\hat{a}_i$. Under the null hypothesis we assume that the second experiment has the same parameterization. We calculate the *p*-value for the difference between the two experiments as the probability that a model with the values $\hat{s}_i$ and $\hat{a}_i$ would produce no counts in the second experiment. So that for $\hat{\mu}_{ij} = T_{ij}\exp(\hat{s}_i)$ with T_{ij} estimated as above within experiment 2 we obtain the following estimate of the *p*-value from equation (1).

$$p_i = \prod_{j=1}^{n} P(N_{ij} = 0|\hat{a}_i, \hat{\mu}_{ij}) = \prod_{j=1}^{n}\left(\frac{1}{1+\hat{a}_i\hat{\mu}_{ij}}\right)^{\hat{a}_i^{-1}} \quad (3)$$

From this we work backwards to come up with approximations for the fold change, confidence interval and prevalence within experiment 2. We start by assuming that we have a situation similar to the one we had when calculating the *p*-value for the case in which both experiments had positive numbers of counts, and denoting the unknown prevalence in experiment 2 by t_i. The test statistic would be

$$Z_i = \frac{\hat{s}_i - \hat{t}_i}{\sqrt{\mathrm{Var}(\hat{s}_i) + \mathrm{Var}(\hat{t}_i)}}$$

Which would be asymptotically distributed as a standard normal. Since we estimated the *p*-value in equation 3, we can write,

$$Z_i \approx \Phi^{-1}(p_i)$$

If we make the additional assumption that $\mathrm{Var}(\hat{s}_i) = \mathrm{Var}(\hat{t}_i)$, we calculate that

$$\hat{t}_i \approx \hat{s}_i - \Phi^{-1}(p_i)\sqrt{2\mathrm{Var}(\hat{s}_i)}$$

From this we can calculate all of the remaining statistics as in the previous case.

REFERENCE

Quantitative PCR One microgram of total RNA was converted to cDNA using iScript cDNA Synthesis Kit (Bio-Rad, 170-8890) in a 20 µl reaction. cDNA was then diluted to 1:5 in H_2O and 2 µl was used in each 20 µl real-time PCR reaction (QuantiTect SYBR Green PCR Kit, Qiagen, 204143). Quantification was performed in triplicates by ViiA7 Real-Time PCR System (Applied Biosystems). Each mRNA expression level was normalized by that of GAPDH. The QuantiTect primers IKBKE (QT00040201), CHEK1 (QT00006734), GAPDH (QT00079247) were purchased from Qiagen.

P53 sequencing Total RNAs were isolated from A2780, HeyA8, Ovcar8, and Igorv1 using RNeasy mini kit (QIagen). cDNA was synthesized as described above. p53 gene was amplified using TaKaRa LA Taq kit with p53 specific primers;

Forward: CTTCCCTGGATTGGCAGCCAGACTG

Reverse: CCCAAAATGGCAGGGGAGGGAGAG.

PCR products were purified by QIAquick PCR purification kit (Qiagen) and the entire p53 was sequenced at both directions using 4 primers including forward, middle forward, middle reverse, and reverse. The sequences of the middle primers are following;

middle-forward: GTCTGGCCCCTCCTCAGCAT CTTAT

middle-reverse: GTGATGATGGTGAGGATGGGC CTCCG

SUPPLEMENTARY FIGURES AND TABLES

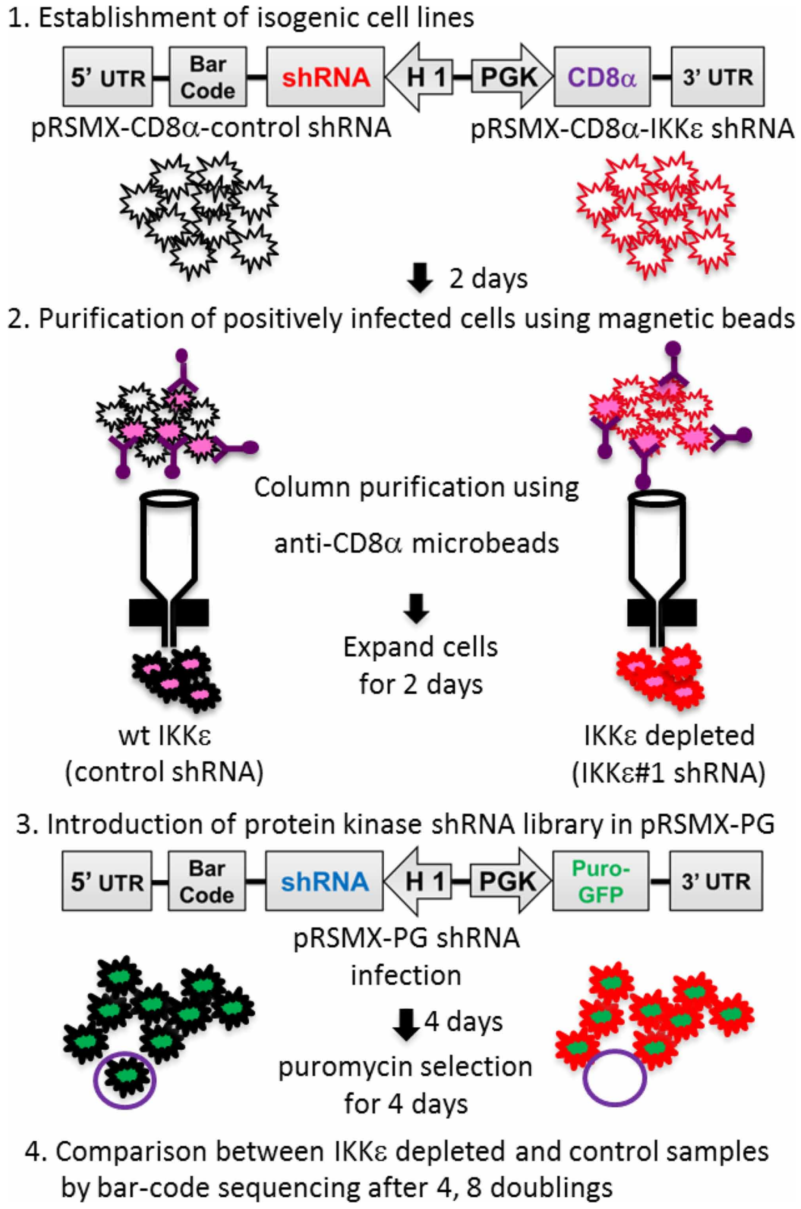

Supplementary Figure S1: Human Kinome shRNA screening in shRNA-mediated IKKε matched ovarian cancer cells **A.** Schematic diagrams of dual shRNA screening steps and time frames are shown. Either IKKε #1 shRNA or negative control shRNA ($E_{1/2}$) of pRSM-LYT2 (CD-8α) was stably infected to establish IKKε isogenic cell lines. $E_{1/2}$ shRNA contains IKKε E1 shRNA sequences without its complementary sequences. pRSMX-PG contains a puromycin resistant gene fused to GFP. This vector is utilized to express shRNA library. Experiments were conducted in 4 replicates. To create a matched cell line pair of IKKε, a bicistronic vector containing either IKKε or negative control shRNA, co-expressed with the transmembrane and extracellular domains of the mouse T-cell surface glycoprotein CD8α, was stably introduced to model IKKε deficiency in ovarian cancer cells. The cell surface expression of mouse CD8α allowed for physical selection of transduced cells, without altering native signaling pathways due to its lack of intracellular signaling domain. Positively transduced cells were isolated using MACS separation columns with mouse CD8α-specific microbeads. In the matched pair of Ovcar5 cells, greater than 94% purity for CD8α positive cells was achieved. After recovery from beads purification, the bicistronic shRNA library containing a puromycin selectable marker fused with GFP was introduced. After second transduction, cells were selected under puromycin allowing approximately two doublings. The purity was 96–99% based on GFP signal at the end of puromycin selection.

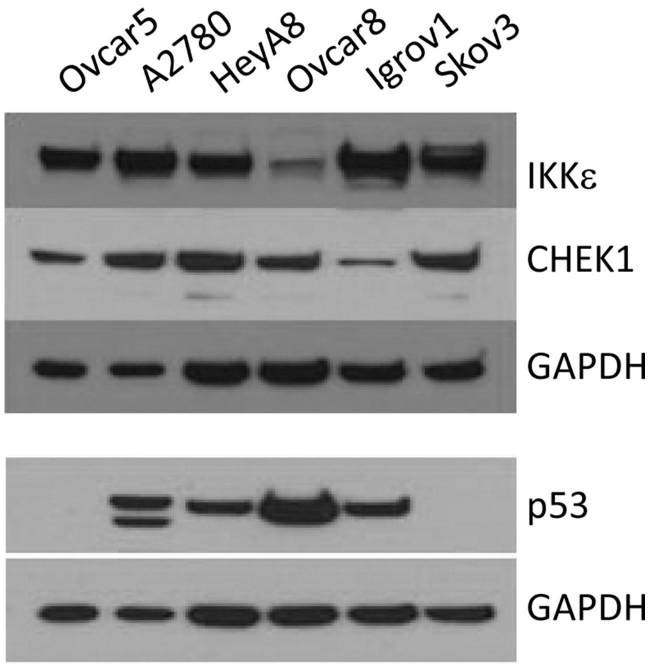

Cell line	p53 status
Ovcar5	null
A2780	wt
HeyA8	P72R
Ovcar8	del (Y126-K132)
Igrov1	Y126C
Skov3	null

Supplementary Figure S2: Expression levels of p53, IKKε, and CHEK1 among 6 ovarian cancer cell lines. The status of p53 was confirmed by sequencing the mRNAs from each cell line. HeyA8 and Ovcar8 carry P72R mutation and 7 amino acids deletions of Y126-132K, respectively, while A2780 and Igrov1 have p53wt and p53Y126C, respectively, as reported previously. Of note, Igrov1 is reported to have CHEK1 (E223fs) mutation although the full length protein was detected. GAPDH was used as a loading control.

	Total Dose (uM)	Fa	CI value
Ovcar5	2.9	0.75	0.52
	1.45	0.63	0.44
A2780	2.9	0.76	1.72
	1.45	0.77	0.83
	0.725	0.46	0.98
HeyA8	2.9	0.68	0.5
	1.45	0.36	0.8
Ovcar8	2.9	0.69	2
	1.45	0.63	1.1
Igrov1	2.9	0.74	2.17
	1.45	0.72	1.16
Skov3	2.9	0.69	1.7
	1.45	0.63	0.97

Supplementary Figure S3: Determination of CI values of IKKε and CHEK1 co-inhibition. CI values for actual experimental points were calculated by CompuSyn from the data as shown in figure 4B.

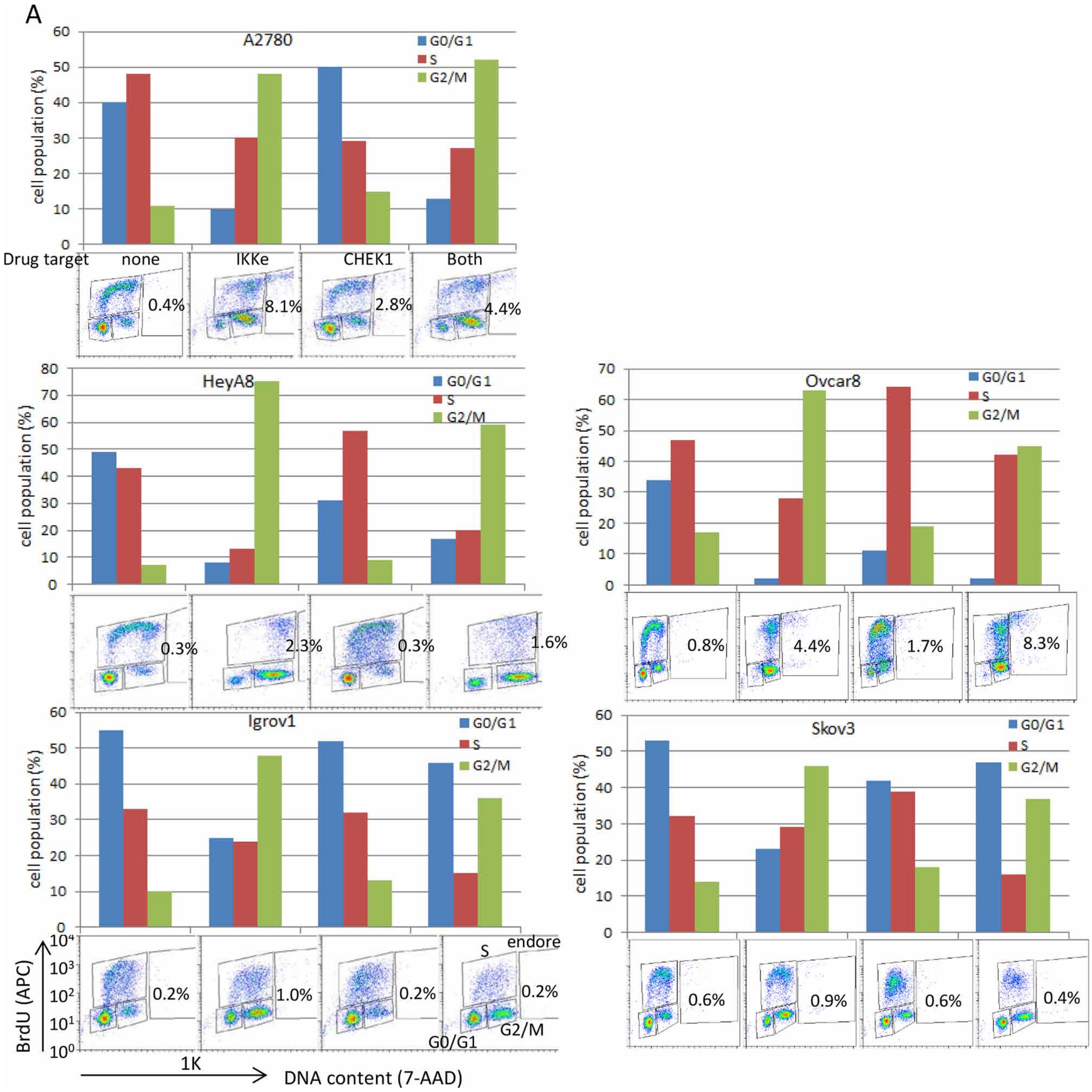

Supplementary Figure S4: Cell cycle analysis after treatment with chemical inhibitors and shRNA knockdowns. **(A)** Cell cycle was analyzed after treatment with IKKε and CHEK1 inhibitors in ovarian cancer cell lines. Cells were seeded at 4-5 × 10^5 per 60 mm plate, 24 hours prior to addition of indicated inhibitors, and treated for 16 hours at final concentration of 2 µM (BX795) and/or 0.5 µM (PF477736) prepared in fresh medium. G_0/G_1, S, and G_2/M phases were measured based on staining of APC-BrdU and 7-AAD by flow cytometry. (*Continued*)

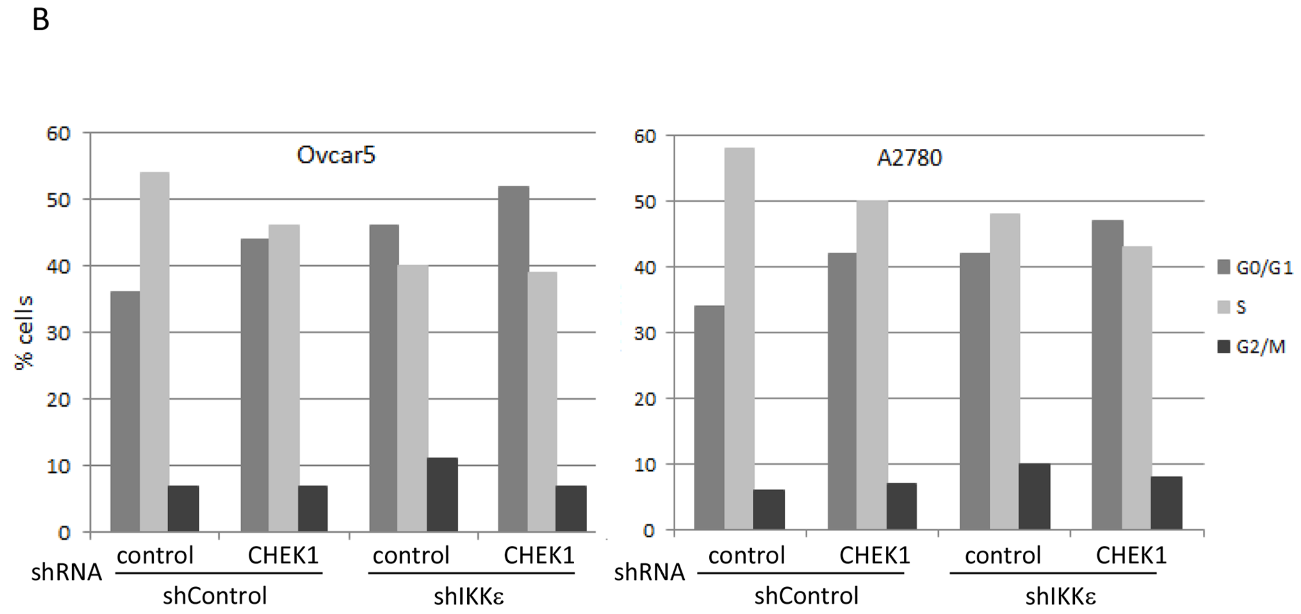

Supplementary Figure S4: (*Continued*) Cell cycle analysis after treatment with chemical inhibitors and shRNA knockdowns. (B) IKKε and CHEK1 double knock-downed cells were selected and maintained in the presence of 25 µg/ml of mycophenolic acid and 2 µg/ml of puromycin. Upon completion of double selection, cells were recovered for 4 days and then seeded 24 hours prior to addition of BrdU. No visible difference among samples in cellular morphology was observed when cells were processed for cell cycle analysis. G_0/G_1, S, and G_2/M phases were measured based on staining of APC-BrdU and 7-AAD by flow cytometry.

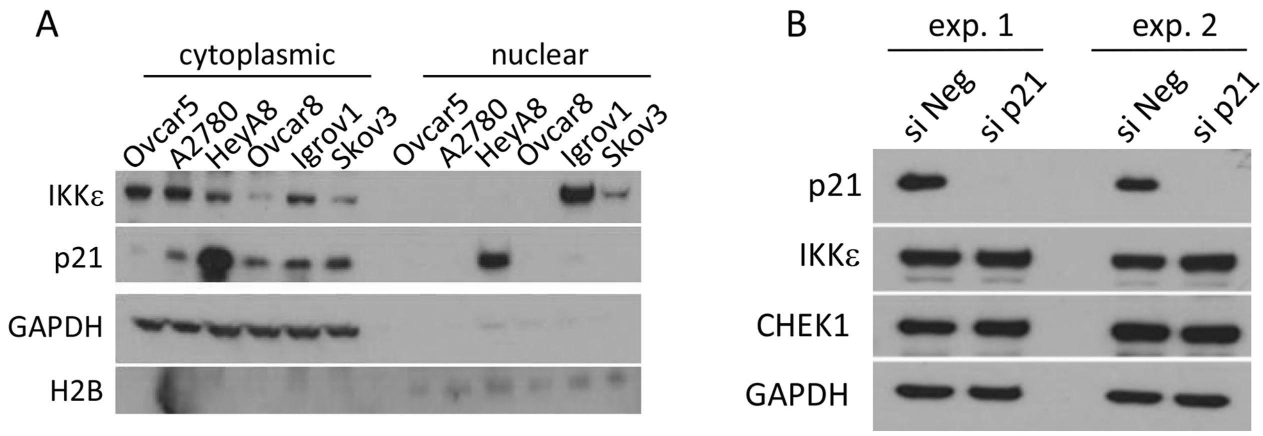

Supplementary Figure S5: Expression of IKKε and p21 in ovarian cancer cells and p21 knockdown in Ovcar5 cells. (A) The ovarian cancer cells were harvested for cytosolic and nuclear fractionation. GAPDH and H2B were used as cytosolic and nuclear markers, respectively. Samples equivalent to 2 × 10^5 cells were loaded per lane. **(B)** The total lysates were prepared at 48 hour post-transfection in 2 independent experiments.

Supplementary Table S1. Sixty five shRNA targets identified by a cut-off with a fold change of ≤ 0.7 with a *p*-value of ≤ 0.05 in Ovcar5

	IKKe depleted vs control		after 4 doublings (at day 7)		after 8 doublings (at day14)	
**pool #**	**Oligo.Name**	**Symbol**	**fold**	***p* value**	**fold**	***p* value**
5	NM_005163.2_bp624_L	AKT1	0.25	0.054	0.08	0.013
1	NM_001184_bp699_L	ATR	0.46	0.012	0.54	0.049
1	NM_001203_bp3832_L	BMPR1B	0.50	0.020	0.47	0.016
5	NM_006888_bp3303_L	CALM1	0.45	0.017	0.33	0.026
1	NM_006549_bp943_L	CAMKK2	0.47	0.048	0.49	0.002
1	NM_003688_bp7687_L	CASK	0.47	0.023	0.47	0.013
1	NM_015076_bp713_L	CDC2L6	0.44	0.018	0.45	0.040
1	NM_001798_bp756_L	CDK2	0.35	0.002	0.40	0.004
1	NM_001260_bp1385_L	CDK8	0.63	0.042	0.21	0.001
1	NM_003948_bp989_L	CDKL2	0.32	0.053	0.36	0.028
1	NM_001274_bp1649_L	CHEK1	0.14	0.002	0.26	0.036
5	NM_004383_bp1621_L	CSK	0.37	0.023	0.42	0.007
1	NM_001348_bp850_L	DAPK3	0.31	0.035	0.36	0.047
1	NM_033403_bp2216_L	DCLK3	0.34	0.002	0.36	0.001
2	NM_006482.2_bp4912_L	DYRK2	0.31	0.001	0.27	0.003
2	NM_006482.2_bp853_L	DYRK2	0.45	0.000	0.42	0.034
2	NM_005233_bp2635_L	EPHA3	0.72	0.013	0.59	0.014
2	NM_004443_bp2198_L	EPHB3	0.50	0.013	0.45	0.000
2	NM_004119_bp622_L	FLT3	0.72	0.001	0.71	0.049
2	NM_182982.2_bp759_L	GRK4	0.35	0.047	0.50	0.012
2	NM_002082.3_bp1642_L	GRK6	0.29	0.034	0.34	0.029
5	NM_016123_bp1786_L	IRAK4	0.27	0.008	0.34	0.032
5	NM_005356.3_bp1260_L	LCK	0.40	0.036	0.27	0.027
2	NM_014916_bp2338_L	LMTK2	0.46	0.003	0.60	0.000
2	NM_001003786_bp1807_L	LYK5	0.45	0.050	0.44	0.002
2	NM_145109.2_bp1911_L	MAP2K3	0.66	0.032	0.34	0.000
2	NM_145160.1_bp1307_L	MAP2K5	0.08	0.026	0.06	0.022
2	NM_002446_bp2833_L	MAP3K10	0.48	0.014	0.37	0.003
2	NM_002446_bp1717_L	MAP3K10	0.52	0.031	0.51	0.000
2	NM_004721_bp3074_L	MAP3K13	0.33	0.001	0.23	0.002
5	NM_003954_bp2687_L	MAP3K14	0.31	0.046	0.21	0.021
5	NM_003954_bp3259_L	MAP3K14	0.35	0.004	0.25	0.005
5	NM_005204_bp2103_L	MAP3K8	0.23	0.001	0.17	0.005
5	NM_005204_bp1954_L	MAP3K8	0.11	0.001	0.21	0.006
5	NM_002746.2_bp578_L	MAPK3	0.21	0.032	0.07	0.006

(*Continued*)

	IKKe depleted vs control		after 4 doublings (at day 7)		after 8 doublings (at day14)	
pool #	Oligo.Name	Symbol	fold	*p* value	fold	*p* value
3	NM_004635_bp468_L	MAPKAPK3	0.35	0.000	0.28	0.003
3	NM_017490.2_bp2722_L	MARK2	0.17	0.026	0.20	0.032
3	NM_005372_bp424_L	MOS	0.47	0.006	0.37	0.023
3	NM_182493_bp2208_L	MYLK3	0.10	0.029	0.07	0.000
3	NM_182493_bp2437_L	MYLK3	0.40	0.001	0.36	0.001
3	NM_012224_bp1053_L	NEK1	0.28	0.010	0.32	0.037
3	NM_005109_bp1767_L	OXSR1	0.56	0.009	0.41	0.001
3	NM_002612_bp3056_L	PDK4	0.53	0.001	0.66	0.026
3	NM_012395_bp1402_L	PFTK1	0.53	0.027	0.50	0.005
5	NM_003557_bp1623_L	PIP5K1A	0.45	0.000	0.39	0.028
5	NM_003558_bp2271_L	PIP5K1B	0.50	0.023	0.39	0.000
5	NM_003558_bp2144_L	PIP5K1B	0.29	0.000	0.42	0.004
3	NM_006256_bp1861_L	PKN2	0.34	0.004	0.46	0.002
5	NM_002661_bp3200_L	PLCG2	0.29	0.013	0.27	0.027
3	NM_001080826_bp1557_L	PRAGMIN	0.23	0.012	0.33	0.000
3	NM_001080826_bp2884_L	PRAGMIN	0.41	0.004	0.61	0.021
3	NM_006252_bp795_L	PRKAA2	0.42	0.007	0.54	0.000
5	NM_002738_bp2428_L	PRKCB	0.41	0.022	0.57	0.047
4	NM_006742_bp441_L	PSKH1	0.59	0.005	0.45	0.009
4	NM_005406_bp2297_L	ROCK1	0.55	0.039	0.74	0.047
4	NM_001006932_bp2227_L	RPS6KA2	0.34	0.027	0.20	0.010
4	NM_003942.2_bp1869_L	RPS6KA4	0.44	0.036	0.21	0.004
4	NM_173354_bp1159_L	SNF1LK	0.46	0.052	0.52	0.049
4	NM_005876_bp5101_L	SPEG	0.51	0.001	0.36	0.023
5	NM_003177_bp1890_L	SYK	0.47	0.047	0.43	0.001
4	NM_004606_bp5721_L	TAF1	0.43	0.008	0.46	0.027
4	NM_016281_bp525_L	TAOK3	0.17	0.016	0.04	0.009
4	NM_007170_bp2826_L	TESK2	0.21	0.000	0.26	0.020
4	NM_012290_bp643_L	TLK1	0.43	0.000	0.63	0.047
4	NM_025195_bp1167_L	TRIB1	0.23	0.029	0.14	0.002
4	NM_021158_bp1557_L	TRIB3	0.30	0.046	0.27	0.018
5	NM_052841_bp1075_L	TSSK3	0.27	0.022	0.41	0.031
5	NM_002822_bp833_L	TWF1	0.43	0.000	0.48	0.007
5	NM_006293_bp1220_L	TYRO3	0.53	0.033	0.45	0.022
5	NM_014683_bp5548_L	ULK2	0.46	0.044	0.17	0.020
5	NM_003390_bp3009_L	WEE1	0.29	0.000	0.41	0.028
5	NM_001079_bp1600_L	ZAP70	0.50	0.002	0.50	0.019

Supplementary Table S2. Primer sequences to PCR-amplify bar-code sequences in shRNA library

Forward primer	AATGATACGGCGACCACCGATCAGGGCAGTGATGTTGCCCCTCGGAAG
Reverse primers	
No1	CAAGCAGAAGACGGCATACGACCTTCTCTAGGCGCCGGAATTAGATCGATCTCTCGAG
No2	CAAGCAGAAGACGGCATACGACCTTCTCTAGGCGCCGGAATTAGATCGATCTCCCGAG
No3	CAAGCAGAAGACGGCATACGACCTTCTCTAGGCGCCGGAATTAGATCGATCTTTCGAG
No4	CAAGCAGAAGACGGCATACGACCTTCTCTAGGCGCCGGAATTAGATCGACCTCTCGAG
No5	CAAGCAGAAGACGGCATACGACCTTCTCTAGGCGCCGGAATTAGATCGATCTCTTGAG
No6	CAAGCAGAAGACGGCATACGACCTTCTCTAGGCGCCGGAATTAGATCGATCCCTCGAG
No7	CAAGCAGAAGACGGCATACGACCTTCTCTAGGCGCCGGAATTAGATCGATTTCTCGAG
No8	CAAGCAGAAGACGGCATACGACCTTCTCTAGGCGCCGGAATTAGATCGGTCTCTCGAG
Rev Primer used in PCR	samples
No1	Ovcar5 B6 Day0-1
No2	Ovcar5 B6 Day0-2
No3	Ovcar5 B6 Day0-3
No4	Ovcar50 B6 Day0-4
No5	Ovcar5 $E_{1/2}$ Day0-1
No6	Ovcar5 $E_{1/2}$ Day0-2
No7	Ovcar5 $E_{1/2}$ Day0-3
No8	Ovcar5 $E_{1/2}$ Day0-4
No1	Ovcar5 B6 Day7-1
No2	Ovcar5 B6 Day7-2
No3	Ovcar5 B6 Day7-3
No4	Ovcar5 B6 Day7-4
No5	Ovcar5 $E_{1/2}$ Day7-1
No6	Ovcar5 $E_{1/2}$ Day7-2
No7	Ovcar5 $E_{1/2}$ Day7-3
No8	Ovcar5 $E_{1/2}$ Day7-4
No1	Ovcar5 B6 Day14-1
No2	Ovcar5 B6 Day14-2
No3	Ovcar5 B6 Day14-3
No4	Ovcar5 B6 Day14-4
No5	Ovcar5 $E_{1/2}$ Day14-1
No6	Ovcar5 $E_{1/2}$ Day14-2
No7	Ovcar5 $E_{1/2}$ Day14-3
No8	Ovcar5 $E_{1/2}$ Day14-4
Rev Primer used in PCR	samples
No1	A2780 B6 Day0-1
No2	A2780 B6 Day0-2

(*Continued*)

No3	A2780 B6 Day0-3
No4	A2780 B6 Day0-4
No5	A2780 $E_{1/2}$ Day0-1
No6	A2780 $E_{1/2}$ Day0-2
No7	A2780 $E_{1/2}$ Day0-3
No8	A2780 $E_{1/2}$ Day0-4
No1	A2780 B6 Day4-1
No2	A2780 B6 Day4-2
No3	A2780 B6 Day4-3
No4	A2780 B6 Day4-4
No5	A2780 $E_{1/2}$ Day4-1
No6	A2780 $E_{1/2}$ Day4-2
No7	A2780 $E_{1/2}$ Day4-3
No8	A2780 $E_{1/2}$ Day4-4
No1	A2780 B6 Day7-1
No2	A2780 B6 Day7-2
No3	A2780 B6 Day7-3
No4	A2780 B6 Day7-4
No5	A2780 $E_{1/2}$ Day7-1
No6	A2780 $E_{1/2}$ Day7-2
No7	A2780 $E_{1/2}$ Day7-3
No8	A2780 $E_{1/2}$ Day7-4
